# Supplementary material for: Learning curve and surgical outcome of robotic assisted colorectal surgery with ERAS program
Source: Sci Rep. 2022 Nov 29;12:20566. doi: 10.1038/s41598-022-24665-w (PMC9709162; doi:10.1038/s41598-022-24665-w)
Supplement: Supplementary file 1 — Supplementary Information 1. [file 41598_2022_24665_MOESM1_ESM.zip › LSC Raw data-Ñ[▒K/052/P_ñw╝╨░O▒Kñσ.pdf]

臺中榮民總醫院 臺中市臺灣大道四段1650號 病理部活體組織[illegible] 臨床醫學實驗及病理診斷中心 檢驗(查)報告

[redacted]

Pathologic diagnosis:
1. Rectum, laparoscopic low anterior resection --- Adenocarcinoma, moderately differentiated. Tumor invaded to perirectal soft tissue, and metastasized to perirectal lymph nodes (2/19). The bilateral cut ends are free of tumor.
2. IMA root, dissection --- Unremarkable lymph nodes (0/2).
3. Rectum, cut end, distal, resection --- Unremarkable.

已電子簽章

Ancillary study for diagnosis:
IHC stains for MLH1, MSH2, PMS2 and MSH6 are done on section C. All above markers are preserved.

敬告：本單僅供醫師參考使用，不具任何申請效力！！
請病患妥為保管，若不慎遺失需自負個人資料外洩責任！

Prognostic and predictive factor:
1. Tumor size: 2x1 cm.
2. Depth of invasion: Perirectal soft tissue.
3. Histologic grade: G2: Moderately differentiated.
4. Angiolymphatic invasion: Not identified.
5. Perineural invasion: Not identified.
6. Tumor deposits (discontinuous extramural extension): Not identified.
7. Surgical Margins: a) Proximal Margin: Uninvolved by invasive carcinoma, distance of tumor from margin: 8.5 cm. b) Distal Margin: Uninvolved by invasive carcinoma, distance of tumor from margin: 3 cm. c) Radial or Mesenteric Margin: Uninvolved by invasive carcinoma, distance of tumor from margin: 1.8 cm.
8. Lymph node status: Involved (2/21).
9. Extranodal involvement: Not identified.
10. Tumor regression grading S/P CCRT: No known presurgical therapy.
11. Type of polyp in which invasive carcinoma arose: Not identified.
12. Additional pathologic findings: Not identified.
13. Pathological TNM Stage: pT3N1b (According to the eighth edition, 2017, American Joint Committee on Cancer Staging Guidelines for Tumors / 2017 CAP guideline).
14. TNM descriptors: Not applicable.

Gross description:
The specimen consists of 1) a segment of colon, 13.5 cm in length and 3.7 cm in proximal cut end circumference, 4.5 cm in distal circumference, and perirectal soft tissue. A gray tan firm annular elevated and centrally ulcerative tumor, 2x1 cm, is noted, 3 cm to the distal cut end. On cross section, the tumor invading to the superficial perirectal soft tissue is suspected with 18 mm to the radial margin. The both cut ends are free of tumor grossly. 2) IMA root, a piece of tan yellow soft tissue, 3.5x2.9x1.2 cm. 3) distal cut end, a piece of tan mucosa-covered soft tissue, 2.5x2x1.1 cm. Representative part for section: A-E and H-K) specimen 1 [A) proximal cut end B-D) tumor E) radial margin H-K) lymph nodes] F) specimen 2 G) specimen 3.

Note:
1. The interpretation of MMR panel and recommended studies are as below: No loss of nuclear expression of MMR proteins: low probability of microsatellite instability-high (MSI-H). Please note: There are exceptions to the above IHC interpretations. These results should not be considered in isolation, and clinical correlation with genetic counseling is recommended to assess the need for germline testing.
2. This case has been peer reviewed by two doctors.

Erratum: on 2019/12/04
Pathologic diagnosis:
1. Rectum, laparoscopic low anterior resection --- Status post neoadjuvant CCRT with residual adenocarcinoma, moderately differentiated. Tumor invaded to perirectal soft tissue, and metastasized to perirectal lymph nodes (2/19). The bilateral cut ends are free of tumor.

Prognostic and predictive factor:
10. Tumor regression grading S/P CCRT: Absent, extensive residual cancer with no evident tumor regression (poor or no response, score 3).
#T-59600_2 #M-81403_2 2 1105 SPER0F
#T-C4520_2 #M-81406_2 2 1105 000000
